# Comparative Transcriptome Analysis Reveals the Molecular Mechanism of *Bacillus velezensis* GJ-7 Assisting *Panax notoginseng* against *Meloidogyne hapla*

**DOI:** 10.3390/ijms242417581

**Published:** 2023-12-18

**Authors:** Wentao Wu, Jingjing Wang, Zhuhua Wang, Xirui Yan, Yang Wang, Xiahong He

**Affiliations:** 1 State Key Laboratory for Conservation and Utilization of Bio-Resources in Yunnan, College of Plant Protection, Yunnan Agricultural University, Kunming 650201, China; wentao_wu121@163.com (W.W.);; 2 Key Laboratory of Forest Resources Conservation and Utilization in the Southwest Mountains of China Ministry of Education, Southwest Forestry University, Kunming 650224, China

**Keywords:** *Panax notoginseng*, *Meloidogyne hapla*, *Bacillus velezensis*, interaction, transcriptome sequencing, lignin-synthesis-related gene, resistance-related gene

## Abstract

The rhizosphere bacteria *Bacillus velezensis* GJ-7, as a biological control agent (BCA), has significant biological control effects on *Meloidogyne hapla*, and has strong colonization ability in the root of *Panax notoginseng*. In this study, we conducted a comparative transcriptome analysis using *P. notoginseng* plant roots treated with *B. velezensis* GJ-7 or sterile water alone and in combination with *M. hapla* inoculation to explore the interactions involving the *P. notoginseng* plant, *B. velezensis* GJ-7, and *M. hapla*. Four treatments from *P. notoginseng* roots were sequenced, and twelve high-quality total clean bases were obtained, ranging from 3.57 to 4.74 Gb. The Gene Ontology (GO) classification and Kyoto Encyclopedia of Genes and Genomes (KEGG) pathway enrichment showed that numerous DEGs are involved in the phenylpropane biosynthesis pathway and the MAPK signaling pathway in the roots of *P. notoginseng* with *B. velezensis* GJ-7 treatments. The analysis results of the two signaling pathways indicated that *B. velezensis* GJ-7 could enhance the expression of lignin- and camalexin-synthesis-related genes in plant roots to resist *M. hapla*. In addition, *B. velezensis* GJ-7 could enhance plant resistance to *M. hapla* by regulating the expression of resistance-related genes and transcription factors (TFs), including ETR, ERF, ChiB, WRKY22, and PR1. The expression of plant disease resistance genes in the roots of *P. notoginseng* with different treatments was validated by using real-time quantitative PCR (qRT-PCR), and the results were consistent with transcriptome sequencing. Taken together, this study indicated that *B. velezensis* GJ-7 can trigger a stronger defense response of *P. notoginseng* against *M. hapla*.

## 1. Introduction

Sanqi (*Panax notoginseng* (Burk.) F. H. Chen) is one of the most important Chinese herbs due to its great medicinal value [1,2]. However, the production of *Panax notoginseng* is seriously affected by soil-borne disease caused by *Meloidogyne hapla*, which can cause significant yield and economic losses [3]. The root-knot nematode (*Meloidogyne* spp.) is one of the most important obligate plant nematode species (PPNs) that can infect host plant roots and induce root galling, thus preventing plant growth and causing complex diseases in synergy with other soil-borne pathogens [4,5]. Root-knot nematode disease is not only limited to medicinal plants, but also found in many other plants, such as agriculture crops and vegetables [3,6]. PPNs cause losses of more than USD 150 billion worldwide every year, most of which are due to *Meloidogyne* spp. [7,8,9,10]. In addition, the restrictions on production caused by *Meloidogyne* spp. are one of the principal reasons for the misuse and overuse of chemical pesticides [11,12]. However, these chemicals have caused serious negative impacts on the eco-environment, animals and humans, and have been restricted in many countries [13,14,15]. Therefore, effective, non-chemical, and environmentally friendly control strategies to manage root-knot nematodes are urgently needed.

Biological control strategies of soil-borne diseases have been studied for more than 100 years and are considered feasible alternative options to chemical control [16]. As an important biological control agent (BCA), bacteria have broad application prospects in the control of soil-borne diseases [17]. Present studies have shown that beneficial bacteria, such as *Bacillus cereus* [18,19,20], *Bacillus altitudinis* [21], *Bacillus velezensis* [2], and *Pseudomonas putida* [19,22], could be used as effective and eco-friendly biocontrol agents to control root-knot nematodes disease. In recent years, the research on the biological control of *Meloidogyne* spp. has been committed not only to the discovery of new BCAs, but also to their biocontrol mechanisms. However, researchers have mainly focused on the direct biocontrol mechanisms of BCAs, including the production of secondary metabolites with nematicidal activity [23], rapid colonization [18,20], and competition for ecological niches [24]. Therefore, the response of host plants to *Meloidogyne* spp., BCAs, and their interactions were ignored.

In previous studies, we obtained *B. velezensis* GJ-7 from the rhizosphere soil of *P. notoginseng*, which can colonize stably and is resistant to *M. hapla* [2], but little is known about how *P. notoginseng* after GJ-7 colonization responds to the infection of *M. hapla.* In the present study, we aimed to reveal the molecular mechanisms of in *P. notoginseng* roots’ response to *B. velezensis* GJ-7, *M. hapla*, and their combined colonization, using an RNA-seq technique.

## 2. Results

### 2.1. Quality Evaluation of RNA-Seq Data 

Evaluation of the quality of the sequencing data for each sample indicated that a total of 113,342,296, 129,511,492, 112,752,838 and 116,755,870 clean reads were generated from the mRNA of Ck, Bcv, Mh and BcvMh treatment groups after removing the low-quality reads and adaptors, which accounted for 93.3%, 93.0%, 92.8% and 93.2% of raw reads, respectively (Supplementary Table S1). The quality values of Q20 in the raw reads were all higher than 98.35%, and likewise the quality values of Q30 in the raw reads were higher than 94.8%. Together, these results demonstrated the high quality and reliability of our sequencing data, and can be used for further analysis.

### 2.2. Identification and Hierarchical Cluster Analysis (HCA) of Differentially Expressed Genes (DEGs) 

To reveal the molecular mechanism of *B. velezensis* GJ-7 assisting *P. notoginseng* against *M. hapla*, two comparative groups were established using sequencing data from 12 libraries. One group used *B. velezensis* GJ-7 treatment samples alone (Bcv) compared with sterile distilled water treatment samples (Ck), and the other group used *B. velezensis* GJ-7 pretreated samples for 3 days and then challenged the plants with *M. hapla* (BcvMh) compared with *M. hapla* treatment samples alone (Mh). It was observed that whether *B. velezensis* GJ-7 was treated alone or pretreated before inoculating the *M. hapla*, a large number of gene expression levels changed in *P. notoginseng* roots. The comparative results revealed that 2524 significant DEGs (1461 downregulated and 1063 upregulated) were identified in the samples of the Bcv treatment (Supplementary Figure S1A), and 3388 significant DEGs (1860 downregulated and 1528 upregulated) were identified in the samples of the BcvMh treatment, respectively (Supplementary Figure S1B). 

**Figure 1:**
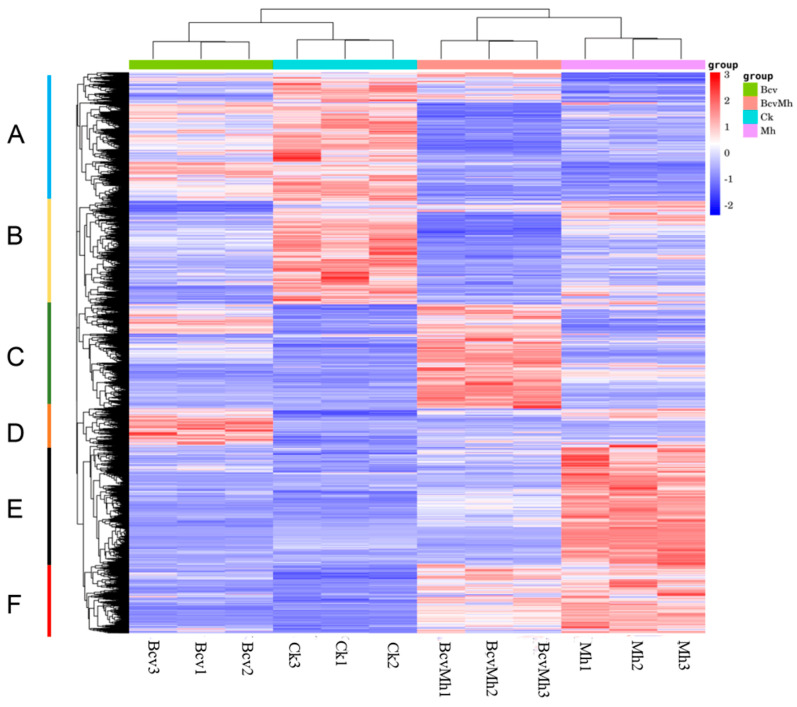
Hierarchical clustering analyses of all DEGs. The analysis was based on four treatments’ FPKM value from the transcriptomic data of CK, Bcv, Mh and BcvMh treatments; red indicates high expression of genes, while blue indicates low expression of genes.

In addition, an HCA (hierarchical cluster analysis) was constructed by using DEGs with significant changes in expression. The analysis results indicate that Ck and Bcv were significantly clustered in the same branch, while Mh and BcvMh were completely clustered in another branch (Figure 1). Based on the expression levels of the DEGs, the gene expression patterns were divided into six groups under four experimental conditions. Cluster A and B contained transcripts that were significantly upregulated in the Ck treatment group. Cluster C contained transcripts that were significantly upregulated in the BcvMh treatment group. Cluster D contained transcripts that were significantly upregulated in the Bcv treatment group. Cluster E and F contained transcripts that were highly upregulated in the Mh treatment group. These results indicated that *P. notoginseng* has different response pathways to the colonization of *B.velezensis*, the infection of *M. hapla*, and their combined effects. 

### 2.3. GO and KEGG Enrichment Analyses of DEGs

**Figure 2:**
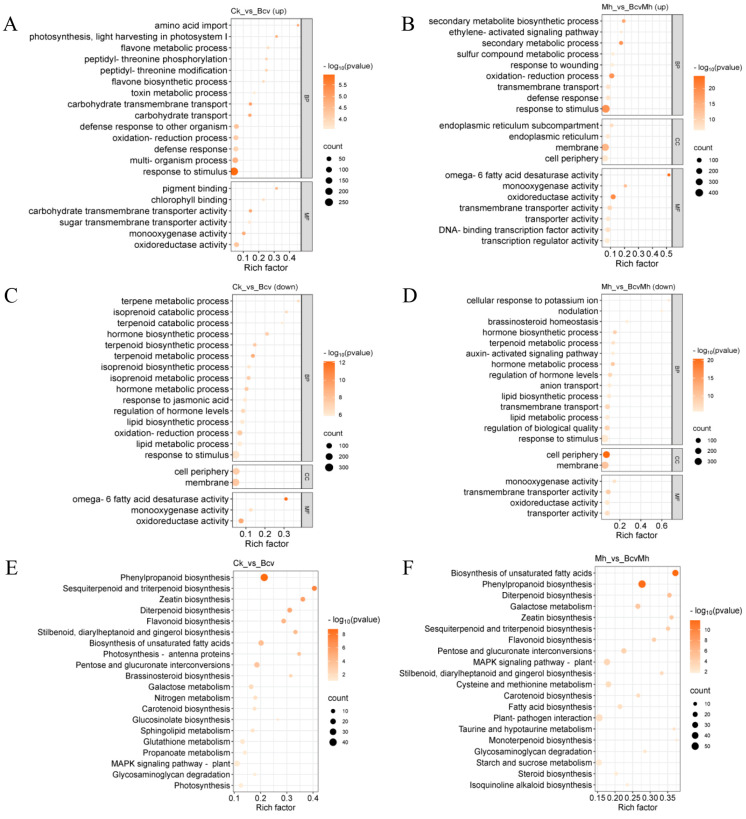
GO and KEGG enrichment analyses of different comparison groups. (**A**) GO enrichment of upregulated genes in Bcv compared with CK; (**B**) GO enrichment of upregulated genes in BcvMh compared with Mh; (**C**) GO enrichment of downregulated genes in Bcv compared with CK; (**D**) GO enrichment of downregulated genes in BcvMh compared with Mh; (**E**) KEGG enrichment of differentially expressed genes in Bcv compared with CK. (**F**) KEGG enrichment of differentially expressed genes in BcvMh compared with Mh.

To reveal the biological functions of DEGs, GO functional enrichment analysis was conducted on DEGs. This study selected the top 20 GO terms that were significantly enriched (*p* < 0.05) for display and analysis. GO enrichment analysis demonstrated that the upregulated genes from Bcv compared with Ck were enriched primarily in the “biological process (BP): defense response (GO:0006952 and GO:0098542), oxidation–reduction process (GO:0055114), flavone metabolic and biosynthetic process (GO:0051552 and GO:0051553)”, and “molecular function (MF): monooxygenase activity (GO: 0004497), transmembrane transporter activity (GO:0015144 and GO:0051119)” (Figure 2A). The upregulated genes from BcvMh compared with Mh were enriched primarily in the “BP: defense response (GO:0006952), oxidation–reduction process (GO:0055114), transmembrane transport (GO:0055085), secondary metabolic process (GO:0019748 and GO:0044550), ethylene-activated signaling pathway (GO:0009873)” and the “MF: oxidoreductase activity (GO:0016491), monooxygenase activity (GO: 0004497)”, and the “cellular component (CC): membrane (GO:0016020), endoplasmic reticulum (GO:0005783)” (Figure 2B). In addition, the GO functional enrichment of down-regulated genes in two comparative groups is mainly concentrated in transmembrane transport and secondary metabolic processes (Figure 2C,D). The above results indicate that *B. velezensis* GJ-7 can improve the expression of defense response genes and the synthesis of secondary metabolites in *P. notoginseng*.

In addition, pathway enrichment analysis with DEGs can provide guidance to identify significant metabolic pathways. To further investigate the biochemical pathways of these DEGs, we mapped all DEGs identified in the RNA sequencing to terms in the KEGG database. KEGG pathway enrichment analysis demonstrated that the DEGs in the comparison of Ck and Bcv were enriched primarily in the “Phenylpropanoid biosynthesis (ko00940)” and “MAPK signaling pathway: plant (ko04016)” pathways (Figure 2E). DEGs in the comparison of Mh and BcvMh were enriched primarily in the “biosynthesis of unsaturated fatty acids”, “phenylpropanoid biosynthesis (ko00940)”, “plant–pathogen interaction” and “MAPK signaling pathway: plant” pathways (Figure 2F). The results indicated that *B. velezensis* GJ-7 treatment could stimulate the expression of more defense-related genes and transcription factors, thereby regulating disease-resistance-related signaling pathways.

### 2.4. Differential Gene Expression Analysis of the Phenylpropane Biosynthesis Pathway

**Figure 3:**
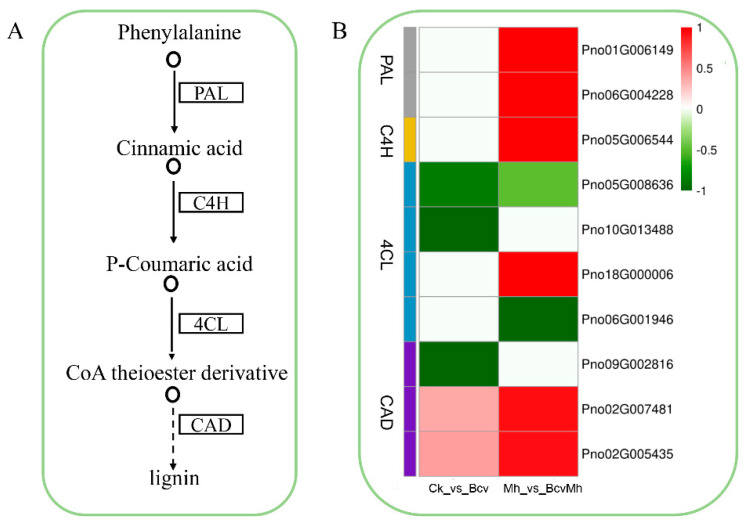
Effect of different treatments on lignin synthesis gene expression. (**A**) Schematic diagram of lignin synthesis pathway; (**B**) expression level of lignin synthesis genes in Bcv and BcvMh treatments.

The above analysis results indicated that the treatment with *B. velezensis* GJ-7 (Bcv and BcvMh) could regulate the expression of genes related to the phenylpropane biosynthesis pathway and participate in lignin biosynthesis. Based on previous studies, phenylalanine ammonia-lyase (PAL), cinnamate 4-hydroxylase (C4H), 4-coumarate CoA ligase (4CL), and cinnamyl alcohol dehydrogenase (CAD) are the four key enzymes involved in the synthesis of lignin (Figure 3A). In the *B. velezensis* GJ-7 treatment samples alone (Bcv), the genes encoding the 4CL protein were downregulated. In addition, partial genes encoding CAD were also downregulated (Figure 3B). These results indicated that the colonization of *B. velezensis* GJ-7 has an inhibitory effect on the synthesis of lignin in the roots of *P. notoginseng*, which is conducive to the formation of a mutually beneficial symbiotic relationship between *B. velezensis* GJ-7 and *P. notoginseng*. In the combination sample (BcvMh), the most critical lignin biosynthesis genes encoding PAL, C4H, 4CL and CAD were upregulated (Figure 3B). This result indicated that pre-colonization of *B. velezensis* GJ-7 might lead to an increase in cell wall lignification to help *P. notoginseng* cope with *M. hapla* infection.

### 2.5. Differential Gene Expression Analysis of MAPK Signaling Pathway

**Figure 4:**
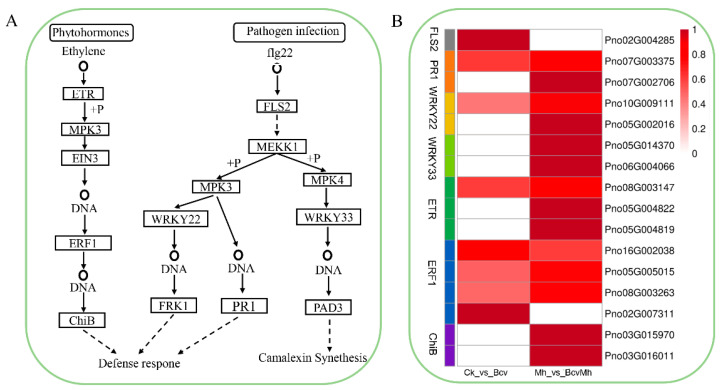
Effect of different treatments on MAPK signal pathway gene expression. (**A**) Schematic diagram of defense response to pathogen infection and phytohormones pathway in MAPK signaling pathway; (**B**) Expression levels of pathogen infection and phytohormone pathway-related genes in Bcv and BcvMh treatments.

Previous studies have demonstrated that the many DEGs are associated with the MAPK signaling pathway in response to *B. velezensis* GJ-7 colonization with or without *M. hapla* infection. Therefore, the above genes involved in the MAPK signaling pathway were analyzed, and the results suggested that *B. velezensis* GJ-7 could affect the expression of numerous MAPK signaling pathway-related genes involved in plant responses to pathogen infection and phytohormone transduction (Figure 4A). In the pathogen infection, the treatment with *B. velezensis* GJ-7 (Bcv and BcvMh) upregulated transcription factor WRKY22 and PR1-related genes to initiate the defense response. In addition, the combination treatment (BcvMh) upregulated transcription factor WRKY33-related genes to active camalexin synthesis in response to the infection of *M. hapla* (Figure 4B). In the phytohormone transduction, the expression of genes related to the ET-mediated pathway was regulated. The treatment with *B. velezensis* GJ-7 (Bcv and BcvMh) upregulated a large number of ethylene receptor (ETR)- and ethylene-responsive transcription factor (ERF)-related genes to activate chitinase synthesis (Figure 4B).

### 2.6. Verification of RNA-seq Data via qRT-PCR

**Figure 5:**
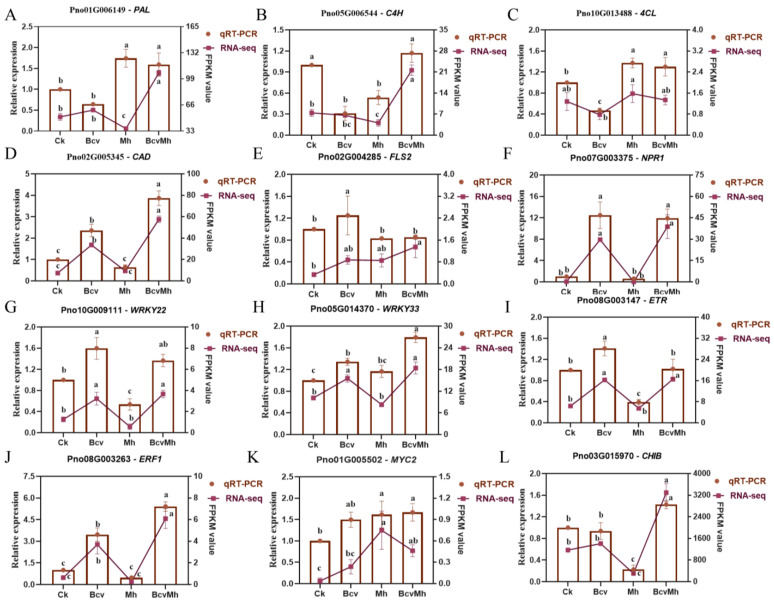
Relative expression of 12 DEGs (**A**–**L**) in *Panax notoginseng* among four different treatments according to qRT-PCR. Ck: The *P. notoginseng* root samples treated with sterile distilled water; Bcv: The *P. notoginseng* root samples treated with *B. velezensis* GJ-7; Mh: The *P. notoginseng* root samples treated with *M. hapla*; BcvMh: The *P. notoginseng* root samples treated with *B. velezensis* GJ-7 and *M. hapla*. The data are shown as means ± SEMs. Different lowercase letters indicate significant differences among the treatments according to a one-way ANOVA and Duncan’s multiple range test (*p* < 0.05).

To confirm the expression patterns identified by the transcriptome sequencing data of genes that were involved in the phenylpropane biosynthesis and MAPK signaling pathway, the transcript levels of 12 genes in the four treatments were examined using qRT-PCR. All the genes selected in this study were successfully amplified, and the patterns of gene expression detected via qRT-PCR were basically consistent with those from the transcriptome sequencing data (Figure 5). Therefore, the DEGs and gene expression profiles from the transcriptome sequencing data were reliable. 

## 3. Discussion 

Transcriptome sequencing technology can comprehensively analyze the changes in all mRNA of plants under biotic and abiotic stress, thereby providing a more accurate and comprehensive analysis of the differential genes and functions obtained [25]. In this study, we utilized RNA-seq technology to investigate the gene expression changes in *P. notoginseng* under the action of *B. velezensis*, *M. hapla*, and their combination. The results showed that inoculation with *B. velezensis* alone only caused significant changes in a small number of genes in *P. notoginseng*, while *B. velezensis* colonization with *M. hapla* infection resulted in significant changes in a large number of genes in *P. notoginseng*. This indicates that the colonization of *B. velezensis* can cause plants to enter a state of “defense priming”, in which plants in this state are infected by pathogens and quickly exhibit resistance reactions [26,27]. 

Induced systemic resistance (ISR) is an important disease resistance mechanism; numerous studies have shown that plant-growth-promoting rhizobacteria (PGPR) can induce ISR to enhance plant defense against various pathogens and insects [28]. However, the role of rhizosphere bacteria in providing ISR against plant parasitic nematodes has not been widely studied. ISR triggers JA- and ET-mediated signaling pathways and enhances disease resistance against pathogen attacks [28,29,30]. In this study, the colonization of *B. velezensis* upregulated the expression of ethylene receptor ETR and ethylene-responsive transcription factor ERF1-related genes, indicating that the *B. velezensis* activated the ET-mediated signaling pathway of *P. notoginseng*. These results indicate that the resistance of *P. notoginseng* plants to *M. hapla* induced by *B. velezensis* is related to the activation of the ET-mediated signaling pathway, which enhances the plant’s disease resistance through ISR. In addition, the colonization of *B. velezensis* also induced significant upregulation of the disease progression-related protein PR1 (or the gene-encoding PR1 protein), which is a marker protein reflecting systemic acquired resistance (SAR) [31,32]. SAR is a pathogen-induced defense system that can be activated individually or jointly by pattern-triggered immunity (PTI) and effector-triggered immunity (ETI) [33,34]. Previous studies have shown that flg22 can be recognized by the pattern recognition receptor (PRR) FLS 2-BAK 1 in the pathogen- or microbial-related molecular pattern (P/MAMP) system [35,36], which can subsequently activate downstream MKK5, MPK3, and WRKY22 transcription factors and ultimately enhance PTI, leading to increased resistance to bacterial and fungal pathogens [37,38]. In our study, we found that *B. velezensis* could upregulate the expression of genes associated with the FLS2-BAK1 complex, WRKY22, and WRKY33, which implied that *B. velezensis* could activate PTI.

The disease resistance of plants depends not only on the presence and expression level of disease-resistant genes, but also on the secondary metabolites they produce. Phenylpropanoid compounds and their derivatives are important secondary metabolites in plants. Under the stress of adversity, they can shift the carbon flow synthesis pathway towards the phenylpropanoid synthesis pathway, thereby increasing the synthesis and accumulation of compounds such as coumarin, flavonoids, flavonols, lignin, etc., and improving the plant’s resistance to pathogens [39]. In this study, pre-colonization of *B. velezensis* could activate the upregulation of lignin synthesis genes PAL, C4H, CAD, and 4CL, indicating that inoculation with GJ-7 increases lignin synthesis in *P. notoginseng* to resist infection of *M. hapla*. Treatment of *M. hapla* alone can also activate the upregulation of some lignin synthesis genes, indicating that lignin is an important defense measure for host plants against infection of root-knot nematode. Numerous research results have shown that lignin deposition in plant cells can effectively prevent pathogen infection [40]. Lignin can form a physical barrier against the cell wall hydrolases and toxins secreted by pathogenic bacteria, hindering their diffusion and preventing the degradation or destruction of plant cell walls [41]. In addition, camalexin (3-thiazol-20-yl-indole) is a phytoalexin which played an important role in resisting the invasion of pathogens [42,43]. The results of this study indicate that pre-colonization of *B. velezensis* can upregulate the expression of transcription factor WRKY33-related genes, thereby increasing the synthesis of camalexin.

## 4. Materials and Methods

### 4.1. Plant Material, Bacteria, and Nematode inoculum

The seedlings of *P. notoginseng* used in this study were obtained from the Daheqiao farm of Yunnan Agricultural University. The two-leaf stage seedlings from the farm nursery land were transplanted to individual pots containing 200 g sterilized soil, and maintained at 25–28 °C in a greenhouse. The *Bacillus velezensis* GJ-7 were obtained from the rhizosphere soil of healthy *P. notoginseng* plants in the forest in Lancang city (Yunnan, China), and deposited in the Plant Nematode Laboratory of Yunnan Agricultural University. The strain GJ-7 was cultured in LB medium at 220 rpm and 30 °C overnight; the bacteria were collected using centrifugation at 8000 rpm for 5 min at 4 °C and finally resuspended in distilled water until a final concentration of 1.0 × 10^8^ CFU/mL (OD_600_ = 0.5; the concentration of bacteria was 3 × 10^8^ CFU/mL, diluted three times to obtain the final concentration) [19,44]. Egg masses of *M. hapla* were obtained from *P. notoginseng* plants infected with the nematodes, and then incubated in sterile water at 28 °C for 24 h to collect second-stage juveniles (J2s) [45,46].

### 4.2. Root Inoculation and Sampling

**Figure 6:**
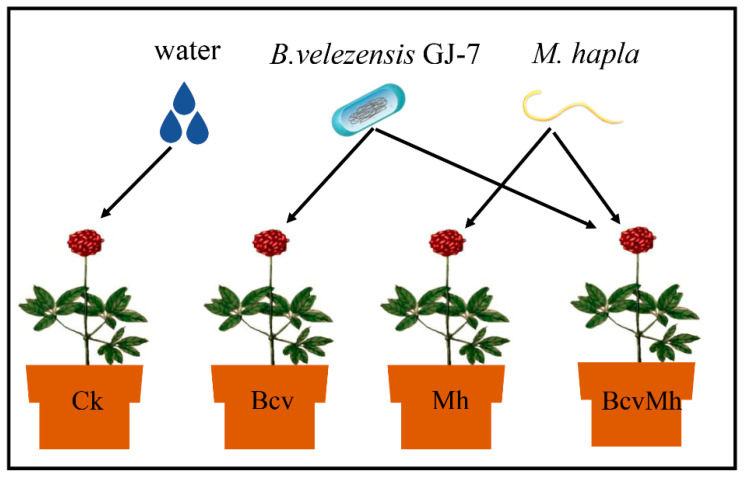
Experimental design of different induction treatments for *Panax notoginseng*. Ck: The *P. notoginseng* root samples treated with sterile distilled water. Bcv: The *P. notoginseng* root samples treated with *B. velezensis* GJ-7. Mh: The *P. notoginseng* root samples treated with *M. hapla*. BcvMh: The *P. notoginseng* root samples treated with *B. velezensis* GJ-7 and *M. hapla*.

After transplanting *P. notoginseng* seedlings for one week, we selected seedlings with consistent growth for the subsequent induction treatment. The experimental design included four treatments (Figure 6). Treatment 1 (Ck): 10 mL sterile distilled water was used to treat seedling roots as a negative control. Treatment 2 (Bcv): The roots of seedlings were inoculated with 10 mL *Bacillus velezensis* GJ-7 inoculum (1.0 × 10^8^ CFU/mL). Treatment 3 (Mh): The roots of seedlings were inoculated with 1500 *M. hapla* second-stage juveniles (J2s). Treatment 4 (BcvMh): The 10 mL strain GJ-7 inoculum was pre-inoculated into the roots of seedlings for three days, and then inoculated with 1500 *M. hapla* second-stage juveniles (J2s). Each treatment included three biological replicates, with each replicate consisting of five *Panax notoginseng* seedlings. After 7 days of inoculation of seedlings with *M. hapla*, the root samples under all treatments were collected, respectively.

### 4.3. RNA-seq of Panax notoginseng Roots and Data Analysis

The total RNA of *P. notoginseng* roots in four different treatments was extracted by using the TRIZOL reagent, and the purity of the RNA was detected using a NanoDrop 2000 instrument (Thermo Scientific, Waltham, MA, USA). The integrity of RNA was determined via agarose gel electrophoresis, and the RNA integrity number (RIN) was determined using an Agilent 2100 instrument (Agilent Technologies, Santa Clara, CA, USA).

The cDNA library construction and sequencing were completed on an Illumina HiSeq^TM^ 4000 platform by Personal Company (Shanghai, China). Clean reads were obtained by removing adapter sequences, along with low-quality sequences, and any reads with more than 10% unknown bases (N). The Q20, Q30 and GC content of the sequences were calculated [47], and clean reads were aligned to the reference genome sequences [48] of *P. notoginseng* using HISAT2 (http://ccb.jhu.edu/software/hisat2/index.shtml, last accessed on 9 November 2022) [49]. All RNA-Seq data generated were saved as FASTQ files, and deposited in the National Center for Biotechnology Information (NCBI) (BioProject accession: PRJNA1022516; BioSample accessions: SAMN37621943, SAMN37621944, SAMN37621945, SAMN37621946, SAMN37621947, SAMN37621948, SAMN37621949, SAMN37621950, SAMN37621951, SAMN37621952, SAMN37621953, and SAMN37621954). The fragments per kilobases per million (FPKM) were used to standardize the levels of gene expression for each replicate sample. The differential expression analysis of each sample was performed using the “DESeq” package (1.10.1) of R, and the differentially expressed genes (DEGs) were determined under the criteria of *p* values < 0.05 and log2(|fold change|) > 1. Volcano plots under different comparison groups were plotted with the R package “ggplots2” and the hierarchical cluster analysis (HCA) was performed with the R package “Pheatmap”.

Functional enrichment analysis of DEGs between samples was performed via GO (Gene Ontology) and KEGG (Kyoto Encyclopedia of Genes and Genomes) using the ClusterProfile package [50]. GO terms and KEGG pathways were considered significantly enriched by DEGs if the *p* values were < 0.05.

### 4.4. Verification of DEGs via Quantitative Real-Time PCR (qRT-PCR) 

To confirm the transcriptome data, the expression levels of 12 DEGs were determined by using qRT-PCR. The first strand of cDNA was synthesized using a PrimeScript^TM^ 1st stand cDNA Synthesis Kit (TaKaRa, Dalian, China). The qRT-PCR was conducted with an ABI 7500 Real-Time PCR System (Applied Biosystems, Waltham, MA, USA) using an AceQ^®^ qPCR SYBR^®^ Green Master Mix (Vazyme, Nanjing, China), and the reaction was performed under the following conditions: 95 °C for 5 min, followed by 40 cycles of 95 °C for 15 s, then 60 °C for 30 s. The qRT-PCR primers used for the DEG validation are listed in Table 1. The *P. notoginseng* encoding actin gene (*PnACT2*) was used as an internal control (CK). To ensure the reliability and reproducibility of the validation results, three independent biological replicates were arranged for each sample. Finally, gene expression was evaluated by applying the 2^−ΔΔCt^ method [51]. 

### 4.5. Statistical Analysis 

The general statistical analysis was carried out using a one-way analysis of variance (ANOVA) with Duncan’s multiple range test (*p* < 0.05) in DPS. Bar charts were drawn with GraphPad Prism 7 (GraphPad Software, San Diego, CA, USA). Heatmap was plotted by https://www.bioinformatics.com.cn (last accessed on 3 December 2023), an online platform for data analysis and visualization.

## 5. Conclusions

This research demonstrates that after colonization in the root of *P. notoginseng*, *B. velezensis* GJ-7 regulated the upregulated expression of lignin and camalexin synthesis-related genes in response to infection by *M. hapla*. In addition, the pre-colonization of *B. velezensis* GJ-7 can improve the expression of defense-related genes to enhance ISR and SAR, thereby improving the health and disease resistance of *P. notoginseng*. In the future, the application of *B. velezensis* GJ-7 could be a way of reducing the severity of plant root-knot nematode diseases. 

## Figures and Tables

**Table 1:** Gene information and primer sequences for qRT-PCR.

Gene ID	Gene Description	Forward Primer (5′-3′)	Reverse Primer (5′-3′)
Pno01G006149	*PAL*	TTGGATGAGGTGAAGCGGAT	CGACAGCTCCACCTTAATGC
Pno05G006544	*C4H*	CCATATCTACAGGCCACGGT	AGGCGTTAACCACCACCTTA
Pno10G013488	*4CL*	CGAGTCAACTGCTGTAGGGA	CACGTAACCAAAGCTCACCC
Pno02G005345	*CAD*	GGGGTTAGGAGGAGTTGGTC	TAGGAATGAATCAGCGCCCA
Pno02G004285	*FLS2*	TGCTCAGCCACAACTACTCA	AACCCAACTGCAGCAAGATG
Pno07G003375	*PR1*	CATGCCCAAAACTCACCACA	GCAGTCTCCAATCCTCGAGT
Pno10G009111	*WRKY22*	CCACAACCATCCAACTCGAC	CTGGAGTCTTTGGGTGTTGC
Pno05G014370	*WRKY33*	ACCACATACGAAGGGAAGCA	GCTGGTGCCCTTGTATTCTG
Pno08G003147	*ETR*	TTGGTCATTGCCTTGTCTGC	ACCACCTTGTTTGCTTGCAA
Pno08G003263	*ERF1*	TTCCACTCCCCAAATTCCGA	TGTGACGAAGCGCCAAATAG
Pno01G005502	*MYC2*	ATGCTGATTACCCGGGTGAA	ACATCCCATCTCCAACTGCT
Pno03G015970	*CHIB*	GAAAACAACCGGGCTGCTTA	GCATTTCCAGCTTGCCCATA
PnACT2	*Actin*	TCCAAGGGTGAATATGATGAATCG	AACCTCTCCAAAGAGAATTTCTGAGT

## Data Availability

The data presented in this study are openly available in National Center for Biotechnology Information (NCBI), BioProject accession: PRJNA1022516.

## Supplementary Materials

The supporting information can be downloaded at: https://www.mdpi.com/article/10.3390/ijms242417581/s1.
