# Supplementary material for: Comparative Transcriptome Analysis Reveals the Molecular Mechanism of Bacillus velezensis GJ-7 Assisting Panax notoginseng against Meloidogyne hapla
Source: Int J Mol Sci. 2023 Dec 18;24(24):17581. doi: 10.3390/ijms242417581 (PMC10743745; doi:10.3390/ijms242417581)
Supplement: Supplementary file 1 [file ijms-24-17581-s001.zip › ijms-2687624-supplementary.pdf]

Supplementary Table S1. Summary of sequences analysis after Illumina sequencing.

Sample	Raw reads	Clean reads	Q20 (%)	Q30 (%)	Total Mapped
Ck1	41149606	38385088	98.55	95.31	36158064 (94.20%)
Ck2	41981484	39213896	98.35	94.83	36838981 (93.94%)
Ck3	38286820	35743312	98.35	95.35	33632896 (94.10%)
Bcv1	50953892	47437402	98.45	95.04	44809341 (94.46%)
Bcv2	45492242	42238798	98.5	95.17	39891316 (94.44%)
Bcv3	42785420	39835292	98.6	95.47	37684903 (94.60%)
Mh1	38975118	35961624	98.43	95	32575581 (90.58%)
Mh2	40401256	37615274	98.45	95.04	34065860 (90.56%)
Mh3	42101092	39175940	98.45	95.06	35532845 (90.70%)
BcvMh1	39012734	36302528	98.44	95.04	33498131 (92.27%
BcvMh2	41943226	39141746	98.35	94.8	36143750 (92.34%)
BcvMh3	44281810	41311596	98.36	94.8	38135948 (92.31%)

Note: Ck: The *P. notoginseng* root samples treated with sterile distilled water; Bcv: The *P. notoginseng* root samples treated with *B. velezensis* GJ-7; Mh: The *P. notoginseng* root samples treated with *M. hapla*; BcvMh: The *P. notoginseng* root samples treated with *B. velezensis* GJ-7 and *M. hapla*; Q20: The percentage of bases in raw reads with a Phred value >20; Q30: The percentage of bases in raw reads with a Phred value >30.

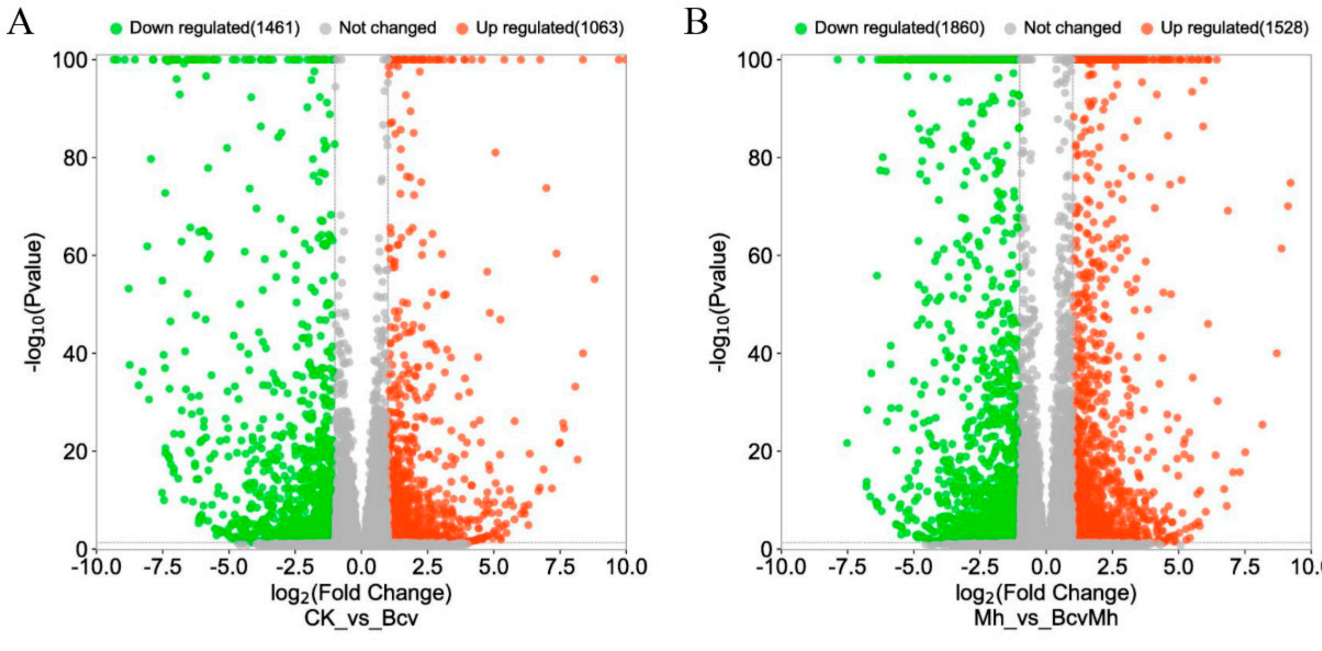

Supplementary Figure S1. Numbers of DEGs in response to *B. velezensis* GJ-7 treatment (**A**) or in combination with *M. hapla* inoculation (**B**). Red color represents up-regulated DEGs, blue color represents down-regulated DEGs.
